# Targeted Depletion of Hyaluronic Acid Mitigates Murine Breast Cancer Growth

**DOI:** 10.3390/cancers14194614

**Published:** 2022-09-23

**Authors:** Vic Zamloot, Nancy Danielle Ebelt, Catherine Soo, Shweta Jinka, Edwin R. Manuel

**Affiliations:** Department of Immuno-Oncology, Beckman Research Institute of the City of Hope, Duarte, CA 91010, USA

**Keywords:** hyaluronic acid, hyaluronan, bacterial hyaluronidase, *Streptomyces koganeinsis*, attenuated *Salmonella typhimurium*, desmoplasia, drug resistance, delivery, breast cancer

## Abstract

**Simple Summary:**

Breast cancer often contains excessive stromal matrix components that serve as a barrier to therapy. One such component is hyaluronic acid (HA), which also plays important roles in tumor progression and spread. In this study, we engineered an attenuated *Salmonella typhimurium* secreting a bacterial hyaluronidase (YS-HAse) and show that it degrades HA within murine breast tumors, resulting in enhanced tumor permeability and growth control. YS-HAse represents a promising new therapeutic approach that could also be used alongside current treatments to improve their delivery and effectiveness.

**Abstract:**

Hyaluronic acid (HA) is highly elevated in breast cancers compared to normal breast tissue and is associated with increased tumor aggressiveness and poor prognosis. HA interacts with cell-trafficking CD44 receptors to promote tumor cell migration and proliferation and regulates both pro- and anti-inflammatory cytokine production through tumor-associated macrophages. The highly negative charge of HA enables its uptake of vast amounts of water that greatly increases the tumor interstitial fluidic pressure, which, combined with the presence of other extracellular matrix components such as collagen, results in tumor stroma with abnormal vasculature, hypoxia, and increased drug resistance. Thus, the degradation of HA in breast cancer may attenuate growth and improve permeability to anticancer agents. Previous methods to deplete tumor HA have resulted in significant off-tumor effects due to the systemic use of mammalian hyaluronidases. To overcome this, we developed a hyaluronidase-secreting *Salmonella typhimurium* (YS-HAse) that specifically and preferentially colonizes tumors to deplete HA. We show that the systemic administration of YS-HAse in immunocompetent murine models of breast cancer enhances tumor perfusion, controls tumor growth, and restructures the tumor immune contexture. These studies highlight the utility of YS-HAse as a novel microbial-based therapeutic that may also be combined with existing therapeutic approaches.

## 1. Introduction

Breast cancer (BC) is the most common cancer type and second-leading cause of cancer-related death in cisgender women and is estimated to constitute 31% of new cases in 2022 [1]. Despite advances in treatment, acquired and inherent therapeutic resistance, as well as metastasis, result in BC, causing 15% of all cancer-related deaths in cisgender women [2]. Thus, novel strategies to target or circumvent resistance and metastatic mechanisms are necessary. The extracellular matrix (ECM) component hyaluronic acid (HA) is a key contributor to both therapeutic resistance and tumor metastasis due to its physical and signaling properties in solid tumors [3,4]. HA is a linear, non-sulfated glycosaminoglycan that is vastly upregulated in BC; pancreatic ductal adenocarcinoma; and lung, ovarian, and colorectal tumors [5]. Its highly negative charge enables its uptake of large amounts of water, which promotes tumoral structural integrity and high interstitial fluidic pressure (IFP) that, together, serve as a formidable therapeutic barrier [6].

In BC tumors, elevated levels of HA are associated with a poorer prognosis due to increased invasiveness, progression, and metastasis [7,8]; indeed, HA greatly composes the invading or “leading” edges of BC tumors [7,9]. HA promotes these characteristics and regulates associated behaviors, such as cell adhesion, motility, and growth in BC, through its interactions with the cell–surface glycoprotein CD44, which is widely expressed in BC tumor cells [10,11]. HA interactions with CD44 can prompt the formation of a complex between CD44 and Erk1/2 that activates MAPK signaling and promotes motility [12,13]. HA-CD44 interactions have also been shown to promote cell migration and invasion through the activation of Rho GTPases, as well as through the recruitment of signaling molecules that activate the PI3K pathway [14]. Notably, these interactions have been shown to upregulate expression of the multidrug resistance receptor and, in turn, promote chemoresistance through activation of the stem cell marker Nanog, as well as through the Stat3 pathway [15].

One manner of disturbing HA–CD44 interactions within tumors is through employing hyaluronidases (HAses). HAses have been used extensively as therapeutics themselves and as spreading agents to facilitate the enhanced dispersal of small molecule-based therapeutics within the ECM [16]. Clinical and commercial HAses are frequently derived from mammalian sources such as bovine or ovine testes or human sperm protein, with several formulations approved by the FDA; however, animal-derived HAses have been noted to cause allergic reactions that prevent repeated administration and adverse reactions due to frequent contamination with proteases and immunoglobulin [17]. The activity of mammalian HAses is additionally not restricted to HA and can result in the degradation of ECM components such as chondroitin and collagen [18]. While additional ECM degradation by HAse may potentially enhance the therapeutic benefits, it may also increase the toxicity observed following the systemic administration of these agents due to the ubiquity of HA and ECM components throughout the body [19]. In addition to safety concerns, the production of these HAses is impaired due to their varied potency and low purity [20]. Although recombinant human HAse has improved on its purification and potency measures, production costs and toxicity remain key issues, with the latter resulting in the reduction of doses in clinical trials to subtherapeutic levels [21,22].

Bacterial HAses have been recently explored as a clinical alternative. In particular, HAse derived from *Streptomyces koganeinsis* has been identified by Messina et al. and confirmed by Pavan et al. to have a similar efficacy but far improved specificity and safety than the available mammalian HAses, as well as stability against proteolytic enzymes [20,23]. These characteristics highlight *S. koganeinsis* HAse as a promising and novel therapeutic agent, but the issue of systemic toxicity has remained largely unaddressed.

One avenue of systemic administration capable of avoiding toxicity is the use of an attenuated *Salmonella enterica*, serovar *typhimurium* vector system. Attenuated *S. typhimurium* (ST) is one of the few microbial agents clinically evaluated in humans and is known for its excellent safety profile, eliciting few adverse effects [24,25]. ST has been shown to naturally and preferentially colonize solid tumors, accumulating at a ratio of 1000:1 to 10,000:1 within tumors compared to a normal liver and spleen [26,27]; its naturally high specificity thus enables the use of ST-based systems for tumor-targeted, systemically delivered therapeutic approaches. Here we employed the well-tolerated ST strain YS1646 (VNP20009) to express the *S. koganeinsis* HAse (YS-HAse), which is tightly controlled by the inducible pBAD plasmid system [24,25,28]. The use of an inducible promoter adds a further layer of protection from systemic toxicity by preventing HAse expression until the addition of a substrate, resulting in a highly tumor-specific and -targeted therapeutic agent that may pose an attractive alternative to the currently available HAse-based therapeutics.

In this study, we developed and characterized YS-HAse, a novel HAse-secreting ST-based agent, and evaluated its effect on murine triple-negative BC (TNBC) tumors. We demonstrate that YS-HAse is able to express and secrete a functional HAse capable of degrading purified and tumor-derived HA in vitro and in vivo, eliciting the control of orthotopic breast tumor growth in a murine model and the elevated intratumoral diffusion of ST. 

## 2. Materials and Methods

### 2.1. Animals and Cell Lines

C57BL/6 mice were obtained from breeding colonies housed at the City of Hope (COH) Animal Research Center (ARC). NOD/SCID gamma (NSG) mice were obtained from Jackson Labs. For all studies, mice were handled according to standard IACUC guidelines under an approved protocol (#17128). EO771, MDA-MB-231, and MDA-MB-436 cell lines were obtained from ATCC^®^ (CRL-3461, HTB-26, and HTB-130). EO771 cells were maintained in RPMI media, and both MDA-MB-231 and MDA-MB-436 cells were maintained in DMEM media. All media was supplemented with 10% FBS, 2 mM L-glutamine, 100 units/mL penicillin, and 100 μg/mL streptomycin. Prior to orthotopic implantation into mice, EO771 and MDA-MB-231 cells were passaged ≤5 times at ≤80% confluency.

### 2.2. YS1646 and Generation of YS-HAse

YS1646 was obtained from ATCC^®^ (202165). YS1646 was cultured in modified LB media (LB-0) containing MgSO_4_ and CaCl_2_ in place of NaCl. The *S. koganeinsis* HAse amino acid sequence (GenBank Accession no. KP313606) was used to synthesize a codon-optimized cDNA (Biomatik; Kitchener, ON, CA) inserted in-frame into a 6xHis-pBAD bacterial expression vector (kind gift from Michael Davidson, Addgene #54762) using XhoI/EcoRI sites. In-frame insertion of HAse into the pBAD vector adds a 6XHis tag to the N-terminus of the protein. YS-LUX was generated using the pAKlux2 plasmid (kind gift from Attila Karsi, Addgene #14080). Plasmids were electroporated into YS1646 using a BTX electroporator (1-mm-gap cuvettes, 1.8 kV, 186 ohms), spread onto LB-0 agar plates containing 100 µg/mL ampicillin, and incubated overnight at 37 °C. Glycerol stocks were generated for pBAD-HAse-positive clones identified by colony PCR and a restriction digest of plasmid preparations.

### 2.3. Bacterial Growth, Viability, and Analysis of HAse Expression

YS-HAse was cultured in media with (induced, IN) or without (uninduced, UN) 0.125–2% (*w*/*v*) L-arabinose at 37 °C, 225 rpm for time intervals ranging from 2 to 24 h. 6XHis-tagged HAse expression was detected in bacterial lysates by Western blot using a primary monoclonal mouse anti-6XHis antibody (Proteintech; Rosemont, IL, USA). Growth kinetics were monitored through absorbance readings at 600 nm (Genesys 30, Thermo Fisher Scientific; Waltham, MA, USA) every 1–2 h up to 24 h. Live/dead staining was performed as previously described [29]. Briefly, YS-HAse was cultured to the logarithmic phase (log phase, 0.7 OD_600_) and UN/IN. Aliquots of bacterial culture were isolated at 3- and 24-h timepoints and centrifuged. The resulting bacterial pellets were stained for 5 min with a 1:1 solution of ethidium bromide and acridine orange in PBS. Pellets were washed of the stain with PBS and resuspended in Fluoromount before mounting on glass slides. Samples were imaged on a fluorescence microscope (Axio Observer 7, Carl Zeiss Inc.; White Plains, NY, USA) with a 63× 1.4NA Plan-Apochromat objective (630× total magnification).

### 2.4. Gel Electrophoresis and Hyaluronic Acid-BSA LB (LB-HA/BSA) Plate Assays

A gel electrophoresis assay to visualize HA degradation was performed as previously described [30]. YS-HAse cultures were grown to 1.5 OD_600_, UN/IN with 2% L-arabinose in LB-0-HA media (LB-0, 100 µg/mL ampicillin and 200 µg/mL purified HA (H-1504, Sigma; St. Louis, MO, USA)), and then incubated overnight in a 37 °C shaker. Cultures were centrifuged, and the supernatant was mixed with glycerol and run on a 0.6% agarose gel at 20 V. The gel was stained with Alcian blue to detect in-gel HA. LB-HA/BSA plates for evaluating HAse activity were generated as previously described [31]. Briefly, LB agar plates containing final concentrations of 0.4 mg/mL HA (H-1504, Sigma; St. Louis, MO, USA), 1% bovine serum albumin fraction V (Sigma; St. Louis, MO, USA), and 100 µg/mL ampicillin were used for plating UN/IN YS-HAse pellets and supernatants (10^6^ colony-forming units (cfu)/5 µL drop) at 37 °C for 2 to 3 days. Plates were then flooded with 1 M glacial acetic acid and rinsed with deionized water. Clear zones were observed against a background of opaque precipitated BSA conjugated to the undigested HA.

### 2.5. Cell Proliferation and Scratch Test Assays

EO771, MDA-MB-231, and MDA-MB-436 cells were assessed for their ability to proliferate when incubated with the degradation products of YS-HAse. YS-HAse was cultured to 1.5 OD_600_ and centrifuged. The resulting bacterial pellets were resuspended in 1 mL of LB-0-HA media with (IN) or without (UN) 2% L-arabinose and cultured overnight. The supernatants were then isolated, and 2 × 10^5^ cells were seeded in each well of a 12-well plate with media (RPMI for EO771 and DMEM for MDA-MB lines) and allowed to attach. All wells were administered 1.5 µg/mL gentamicin (Sigma; St. Louis, MO, USA). Control wells were administered 2% L-arabinose and 300 µL LB-0 with 100 µg/mL ampicillin (Sigma; St. Louis, MO, USA). HA wells were administered the same dose of L-arabinose and 300 µL of LB-0-HA media. UN and IN wells were administered the respective supernatants of the YS-HAse overnight cultures. At 24, 48, and 72 h post-administration, cell counts were performed in triplicate using a TC20 automated cell counter (Bio-Rad; Hercules, CA, USA). For the scratch test assay, the cells were seeded, and the treatments were prepared as mentioned above. Prior to the administration of degradation products and reagents, a p200 pipette tip was used to scratch a vertical line through the adhered cells. Wells were washed gently with PBS to remove detached cells and supplied with DMEM media with 3 µg/mL gentamicin. When the scratches in the control wells were completely covered by adherent cells, all wells were fixed in ice-cold 100% methanol for 10 min, stained with a 0.5% crystal violet solution for 10 min, and rinsed of excess stain using deionized water.

### 2.6. Orthotopic Tumor Implantation and Detection of Tumor Colonization by YS1646

Previously published methods were used for the orthotopic implantation of EO771 or MDA-MB-231 cells into the left fourth (L4) mammary fat pad of C57BL/6 or NSG mice [32]. Briefly, while anesthetized and using sterile techniques, approximately 1 × 10^5^ EO771 cells or 3 × 10^6^ MDA-MB-231 cells, each in a volume of 100 µL PBS, were injected through the skin into the L4 mammary fat pad using a 27-gauge needle. C57BL/6 mice with palpable EO771 tumors (>150 mm^3^) were intravenously (i.v.) injected with 5 × 10^6^ YS-LUX. Actively growing YS-LUX is constitutively bioluminescent and was used to evaluate the YS1646 colonization of EO771 tumors in vivo using intravital imaging (LagoX, Spectral Instruments Imaging; Tucson, AZ, USA). Mice were imaged daily following the administration of YS-LUX, and the bioluminescent signal was evaluated using Aura software (Spectral Instruments Imaging; Tucson, AZ, USA).

### 2.7. Immunohistochemistry/Immunofluorescence (IHC/IF)

EO771 tumor sections from C57BL/6 mice treated i.v. with UN/IN YS-HAse were de-paraffinized, rehydrated, and stained with the 5 µg/mL HA-binding protein (HABP) (385911, MilliporeSigma; Burlington, MA, USA) and 1:200 anti-ST antibody (sc-52223, Santa Cruz Biotechnology; Dallas, TX, USA) overnight. A 1:50 DyLight 594 Streptavidin (SA-5594, Vector Labs; Newark, CA, USA) and 1:50 goat anti-mouse AlexaFluor 680 (A21057, Invitrogen; Waltham, MA, USA) were then used to visualize HA and ST by fluorescence microscopy, in addition to 1:200 DAPI for visualizing the nuclei. IHC staining of murine immune subsets with DAB was performed by the Pathology Research Services Core (City of Hope; Duarte, CA, USA) using anti-F4/80, anti-CD11b, anti-CD4, and anti-CD8 antibodies, with the nuclei visualized via hematoxylin counterstaining. Brightfield and fluorescent tissue images were acquired by tiling on an Axio Observer 7 (ZEN 2.3 Pro (Blue), Carl Zeiss Inc.; White Plains, NY, USA) inverted microscope equipped with a 10× 0.45NA Plan-Apochromat objective (100× total magnification). Fluorescent images of ST were acquired with a 63× 1.4NA Plan-Apochromat objective (630× total magnification). Stitching was performed using ZEN 2.3 Pro (Blue) software (Carl Zeiss Inc.; White Plains, NY, USA). Quantification of the immune subsets was performed using Quantitative Pathology & Bioimage Analysis (QuPath) software (v0.3.2, University of Edinburgh; Edinburgh, UK) [33].

### 2.8. Administration and Induction of YS-HAse in Tumor-Bearing Mice

For all experiments, 6–8-week-old female mice were used. C57BL/6 mice with palpable EO771 tumors (>50 mm^3^) or NSG mice with palpable EO771 or MDA-MB-231 tumors (>50 mm^3^) were i.v. injected with 5 × 10^6^ YS-HAse per day for two days. Intravenous injections were administered to the retro-orbital vein of the right eye. Two days after administering the first dose of YS-HAse, mice were intraperitoneally administered 150 mg L-arabinose or PBS. Tumors were then measured approximately every other day until the endpoint.

### 2.9. Flow Cytometry

One million live cells were counted using trypan blue and first stained with a fixable viability dye (eBiosciences 65-0866-14, Thermo Fisher Scientific; Waltham, MA, USA) for 30 min at 4 °C. Cells were washed in flow wash buffer (PBS with 0.05% sodium azide and 3% FBS) and stained with surface antibodies for 40 min at 4 °C. Cells were washed in a flow buffer and fixed in a flow buffer plus 4% PFA before filtering through 40-μm mesh strainer tubes (BD Biosciences; San Jose, CA, USA). Flow cytometry was performed on the BD Fortessa X20 cytometer, and the data were analyzed using FlowJo Version 10 (Becton, Dickinson & Co.; Franklin Lakes, NJ, USA). Flow cytometry antibodies used from BD Biosciences (San Jose, CA, USA) include: CD45-APC-R700 (565478), CD8-PE (553033), CD4-APC-H7 (5560181), Ly6G-BV605 (563005), Ly6C-FITC (561085), CD11b-APC (561690), PD-1-BV421 (562584), NK1.1-BV650 (564143), and MHCII-PerCPCy5.5 (562363). Flow cytometry antibodies used from eBiosciences (Thermo Fisher Scientific; Waltham, MA, USA) include: F4/80-Super Bright 780 (78-4801-82).

### 2.10. Statistics

All statistical analyses were performed using Prism software by GraphPad (v9) (San Diego, CA, USA). Unless otherwise indicated, all error bars represent the standard error of the mean.

## 3. Results

### 3.1. Construction and In Vitro Characterization of YS-HAse

**Figure 1:**
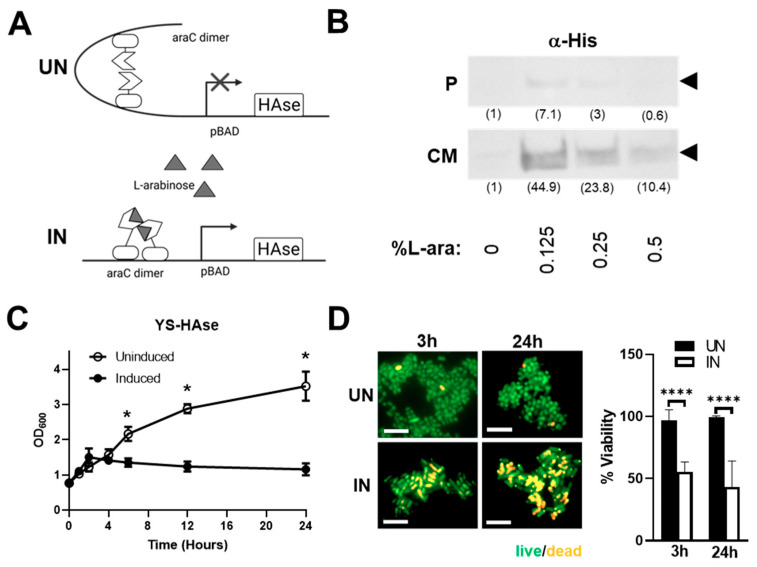
**Induction of HAse expression leads to its secretion by YS-HAse and attenuated bacterial growth.** (**A**) Schematic of the expression of HAse in pBAD under uninduced (UN, top) and induced (IN, bottom) conditions. (**B**) Attenuated YS1646 transformed with the pBAD-HAse construct (YS-HAse) was cultured in growth media until reaching the log phase (0.7 OD_600_) and was uninduced (0% L-arabinose) or induced with 0.125–0.5% L-arabinose (L-ara) for 2 hours at 37 °C. The bacterial cell lysates (P) or concentrated culture media (CM) for each L-ara concentration were run on a polyacrylamide gel and subjected to Western blot analysis against a His tag fused to the amino terminus of HAse (α-His). The relative band density in parentheses (ImageJ) normalized to 0% L-ara. (**C**) The growth curve of YS-HAse post-induction. Optical density readings (OD_600_) for uninduced and induced (2% L-ara) YS-HAse cultures were measured over 24 hours. Uninduced and induced cultures were done in triplicate, and their growth curves are compared. * *p* < 0.05, *t*-test. (**D**) Live/dead staining of uninduced and induced YS-HAse cultures. YS-HAse cultures were grown to the log phase, uninduced (UN) or induced (IN, 2% L-ara), and stained at the indicated timepoints with a 1:1 mixture of acridine orange (live, green) and ethidium bromide (dead, orange) in PBS. Percent viability = (total-dead)/total × 100%. Fluorescent imaging was performed with an Axio Observer 7 (Carl Zeiss) with a 63× objective (630× total magnification). Scale bar = 5 μm. Percent viability of UN and IN YS-HAse is based on 10 random fields. **** *p* < 0.0001, *t*-test.

Constitutive HAse expression can be toxic to live attenuated ST vectors and may cause adverse off-tumor effects in vivo before the recombinant ST has colonized tumor tissue [19,34]. To circumvent this, we employed an inducible pBAD expression system that is tightly regulated by L-arabinose (Figure 1A). The construct includes the araBAD (arabinose) operon and the gene encoding its positive and negative regulator, araC [28]. To generate pBAD-HAse, an ST codon-optimized HAse sequence (based on the amino acid sequence of the well-characterized *S. koganeinsis* HAse) was synthesized and cloned into the pBAD vector. pBAD-HAse was then used to transform the attenuated ST strain YS1646, selected for its excellent tumor-colonizing ability and safety profile in cancer patients [24,25,26,35]. Positive clones were cultured in growth media under uninduced (UN; 0% L-arabinose (L-ara)) or induced (IN; 0.125–0.5% L-ara) conditions and evaluated for the expression of HAse by Western blot analysis (Figure 1B). The IN culture media showed a robust expression of HAse, with a 6.3-fold increase in expression at 0.125% L-ara compared to the pellet lysates, suggesting that YS-HAse predominantly secretes HAse into the culture media.

To assess the impact of HAse expression on the growth of YS1646, we performed growth kinetic culture studies in the absence or presence of L-ara (Figure 1C). YS-HAse was cultured to the logarithmic phase and uninduced or induced. The optical density (OD_600_) readings performed at various timepoints over 24 h show that uninduced YS-HAse grows steadily over time, reaching an OD_600_ of 2.167 at 6 h and 3.520 by 24 h. Conversely, the induction of YS-HAse significantly impairs its growth, resulting in a 3.2-fold reduction in growth at 24 h compared to the uninduced (*p* < 0.05). These data indicate that the expression of HAse is toxic to YS1646, resulting in attenuated growth, death, or both. To help distinguish the cause of reduced growth kinetics, we performed live/dead staining to quantify ST viability following induction. The YS-HAse cultures were grown to the log phase and cultured under UN or IN conditions for 24 h. The samples were taken after 3 and 24 h of culturing, which correlate with previous OD_600_ readings of growth attenuation under IN conditions, and stained with a 1:1 mixture of acridine orange and ethidium bromide to quantify the percentage of viable bacteria. As shown in Figure 1D, UN YS-HAse remained nearly 100% viable over 24 h, whereas IN YS-HAse exhibited a ~50% reduction in viability after 3 h and ~60% reduction in viability after 24 h (*p* < 0.0001). These data suggest that the expression of HAse is toxic to YS-HAse and may therefore compromise its ability to colonize tumors in vivo if HAse were produced constitutively. These results highlight the importance of using an inducible expression system so that systemically administered YS-HAse can first colonize the tumor prior to being induced for HAse secretion.

### 3.2. YS-HAse Secretes Functional HAse following L-Arabinose Induction

**Figure 2:**
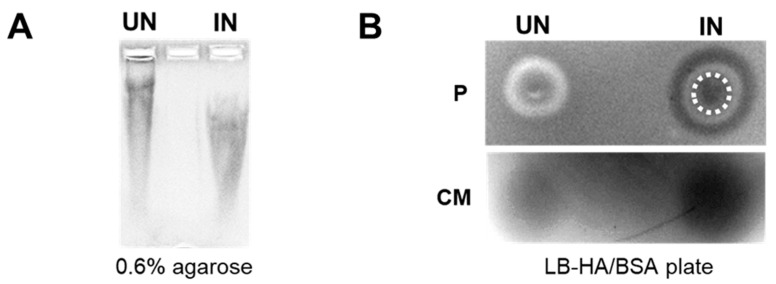
**YS-HAse secretes functional HAse following L-arabinose induction.** (**A**) In-gel degradation assay. YS-HAse cultures were grown to an OD_600_ of 1.5 and UN or IN with 2% L-ara in culture media containing 200 μg/mL HA and then incubated overnight in a 37 °C shaker. The cultures were centrifuged, and the supernatant was mixed with glycerol and run on a 0.6% agarose gel at 20 V. The gel was stained with Alcian blue and destained with acetic acid to detect the HA smear. (**B**) Plate-clearing assay of YS-HAse. YS-HAse cultures were grown to an OD_600_ of 0.7 and UN or IN with 0.125% L-ara. The concentrated pellet (P, top) and isolated and filtered culture media (CM, bottom) were spotted on LB-HA/BSA agar plates and incubated. The plates were then flooded with 1 M glacial acetic acid. The degradation of HA is observed as clear, black areas. A white, dashed circle represents the area of the initial colony spot.

We next performed in vitro studies to evaluate the functionality of the HAse secreted from YS1646. An in-gel degradation assay was used to visualize the shifts in HA substrate cultured with UN or IN YS-HAse. In this assay, YS-HAse was cultured under UN or IN conditions in media containing HA. The supernatants were then collected and run on an agarose gel followed by Alcian blue staining to visualize HA. Digested HA will run faster through the gel compared to undigested HA. Indeed, the supernatant from IN YS-HAse showed a lower shifted HA smear compared to UN YS-HAse (Figure 2A), indicating that HAse secreted by IN YS-HAse has functional enzymatic activity.

We further evaluated the functionality of the secreted HAse via a plate-clearing assay, involving the incubation of YS-HAse on LB agar plates containing HA and BSA (LB-HA/BSA). The exposure of these plates to glacial acetic acid results in the precipitation of BSA and undigested HA, turning the agar an opaque white color with areas of HA digestion remaining clear and dark when imaged. The bacterial pellet (Figure 2B, top) and supernatant (culture media) (Figure 2B, bottom) of UN or IN YS-HAse cultures were isolated, spotted on LB-HA/BSA plates, and incubated at room temperature. The plates were then flooded with acetic acid to visualize areas of HA degradation and washed with deionized water to remove residual ST. As expected, both the colony and supernatant of UN YS-HAse showed no clearing, suggesting no functional HAse was present. However, IN YS-HAse exhibited a robust clearing of the agar encompassing the initial area of the spotted pellet and supernatant, demonstrating the digestion of the HA within the agar. Furthermore, we observed a ring of clearing outside of the induced pellet area, forming a set of concentric circles that indicates secretion of a functional HAse by the spotted cell pellet. Together, these results suggest that YS-HAse expresses and secretes a functional HAse capable of degrading purified HA in vitro.

### 3.3. YS-HAse Degradation Products Do Not Affect BC Cell Proliferation and Migration

**Figure 3:**
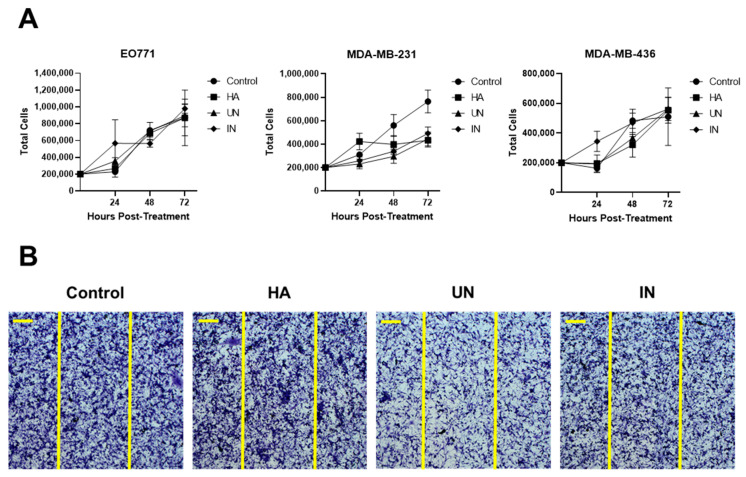
**YS-HAse degradation products do not affect cell proliferation or migration.** (**A**) Proliferation assay of 3 TNBC cell lines. YS-HAse cultures were grown to an OD_600_ of 1.5 and centrifuged. Pellets were resuspended in LB-0 media containing 200 μg/mL purified HA (LB-0-HA, and UN or IN with 2% L-ara overnight at 37 °C. The supernatants were collected and added to 12-well plates seeded with 2 × 10^5^ EO771, MDA-MB-231, or MDA-MB-436 cells. The control wells received media and L-ara; the HA wells received L-ara and LB-0-HA media. All wells received 1.5 μg/mL gentamicin. The cell counts were performed in triplicate at the indicated timepoints. (**B**) The scratch test assay of MDA-MB-231. The MDA-MB-231 cells were seeded and treatments were prepared as in (**A**). When the cells were confluent, a p200 pipette tip was used to make a vertical scratch through the well. Vertical yellow lines represent the initial width of the scratch. When the control well fully closed, all other wells were stained with crystal violet and imaged using brightfield microscopy. Scale bar = 200 μm.

End products generated from HA degradation may potentiate tumor progression, which has been suggested by studies showing increased tumor cell growth and migration following overexpression of the mammalian hyaluronidase HYAL1 [36,37]. We therefore evaluated the effect of HA degradation products, generated by YS-HAse, on cell proliferation and migration in vitro through proliferation and scratch test assays (Figure 3). YS-HAse was cultured and centrifuged to form a concentrated pellet. Bacterial pellets were then resuspended and incubated overnight in LB-0 media containing purified HA (LB-0-HA) under UN or IN conditions. The supernatants were then isolated and added to 12-well plates containing 2 × 10^5^ EO771, MDA-MB-231, or MDA-MB-436 cells per well. The control wells were administered L-ara and LB-0; the HA wells were administered L-ara and LB-0-HA. All wells were given gentamicin to prevent residual ST growth. At 24, 48, and 72 h post-treatment, the cells were counted to assess any changes in their proliferation ability (Figure 3A). Regardless of the cell line or treatment, no significant difference was observed in their proliferation, suggesting that YS-HAse degradation products do not affect cell proliferation. To assess cell migration, 2 × 10^5^ MDA-MB-231 cells were seeded in each well of a 12-well plate and grown to confluency. Using a pipette tip, each well was scratched vertically across the adherent cells, and YS-HAse degradation products and associated reagents were added in the same manner as in the proliferation studies. Following complete closure of the scratch in the control wells, all other wells were fixed and stained with crystal violet to assess the degree of cell migration into the scratch. Regardless of the treatment, all scratches were fully infiltrated (Figure 3B), indicating that YS-HAse degradation products also do not increase cell migration in vitro.

### 3.4. YS-HAse Specifically and Preferentially Colonizes Orthotopic BC Tumors

**Figure 4:**
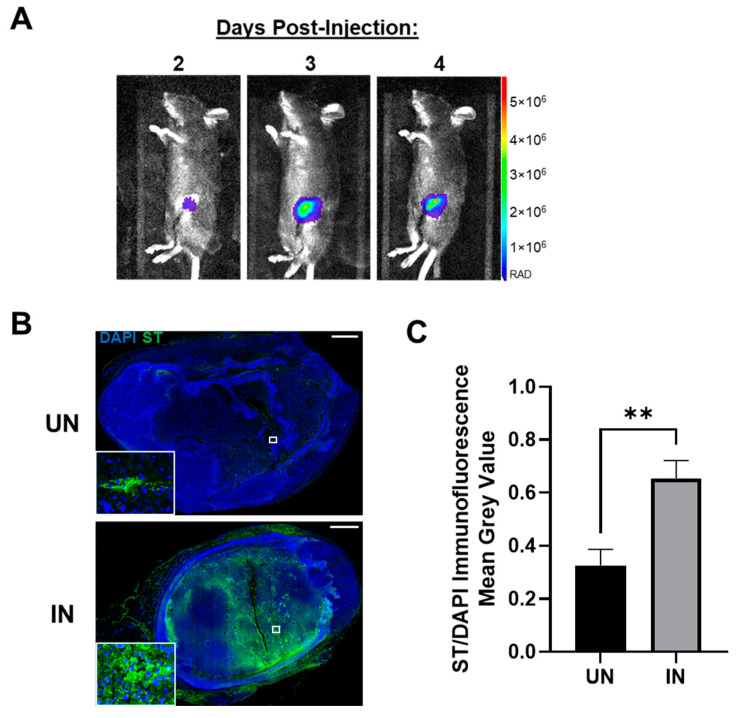
**YS-HAse preferentially and specifically colonizes EO771 tumors in vivo.** (**A**) Tumor colonization by YS1646. C57BL/6 mice bearing orthotopic EO771 tumors (>150 mm^3^) were administered 5 × 10^6^ colony-forming units (cfu) of YS1646 expressing luciferase (YS-LUX) by intravenous (i.v.) injection. Intravital imaging was performed 2, 3, and 4 days post-injection using IVIS. Representative images are shown. (**B**) Immunofluorescence (IF) staining of YS-HAse-treated tumors. C57BL/6 mice bearing orthotopic EO771 tumors (~100 mm^3^) were i.v. administered 5 × 10^6^ cfu of YS-HAse per day over two days (*n* = 5). Forty-eight hours after the first dose, the groups were intraperitoneally (i.p.) administered PBS (UN) or 150 mg L-arabinose (IN) and then euthanized 24 h post-induction. Serial tumor sections were then subjected to IF staining to detect YS-HAse (ST) using the HA-binding protein (HABP) and anti-ST 0–4 primary antibody, followed by fluorescence-conjugated secondary antibodies. All images are representative of ≥3 experiments. Scale bar = 1000 µm. (**C**) The quantification of ST per area was approximated by dividing the mean grey value (MGV) of the ST signal by the MGV of DAPI. The MGV was measured using ZEN Blue software. ** *p* < 0.01, *t*-test.

A key metric contributing to the innovation of YS-HAse is its tumor specificity, as all previous treatments were systemically active and elicited severe adverse, off-tumor toxicity [21,22]. Thus, we further evaluated the ability of YS-HAse to colonize orthotopic EO771 tumors in real time using bioluminescent intravital imaging (IVIS). As YS-HAse is not designed for detection by IVIS, we employed YS1646 transformed with the pAKlux2 plasmid (YS-LUX), which emits a high bioluminescence in live ST [38]. Female C57BL/6 mice bearing orthotopic EO771 tumors (>150 mm^3^) were intravenously (i.v.) administered 5 × 10^6^ colony-forming units (cfu) of YS-LUX. The expression and location of YS-LUX in the mice was evaluated daily via IVIS (Figure 4A). YS-LUX was first detected in mice two days post-administration and was solely concentrated within the tumors. The YS-LUX signal within the tumor increased and reached its peak between days three and four post-administration; this signal waned but was still present at seven days post-administration (Supplementary Figure S1). The YS-LUX expression remained confined to the tumor area, indicating that YS1646 specifically and preferentially colonizes EO771 tumors and that peak colonization occurs 3 to 4 days following its administration.

In addition to the real-time tracking of YS-LUX, we chose to evaluate tumor colonization by YS-HAse under UN and IN conditions. Female C57BL/6 mice bearing orthotopic EO771 tumors (~100 mm^3^) were i.v. administered 5 × 10^6^ cfu YS-HAse per day for two days (1 × 10^7^ cfu total). In accordance with the peak colonization time observed by IVIS (Figure 4A), the mice were intraperitoneally (i.p.) UN or IN on day three. All mice were euthanized on day four, and the tumors were removed and sectioned. Serial sections were subjected to immunofluorescence (IF) staining using the HA-binding protein (HABP) and anti-ST primary antibody to visualize HA and ST, as well as DAPI for the nuclei. IF imaging of the DAPI and ST levels in the UN and IN tumor sections revealed elevated tumor colonization by IN YS-HAse compared to that of UN YS-HAse (Figure 4B). While both UN and IN YS-HAse treatments showed the detectable colonization of EO771 tumors (Figure 4B, inset), the quantification of the total ST signal per total tumor area (approximated by the total DAPI signal) demonstrated a significant 2.004-fold increase in colonization by IN YS-HAse compared to UN YS-HAse (*p* < 0.01) (Figure 4C). The greater tumor colonization achieved by IN YS-HAse suggests that induction, and thus the production of HAse, allows ST to diffuse further throughout the tumor, most likely as a result of intratumoral HA degradation.

### 3.5. YS-HAse Degrades Intratumoral HA in Murine BC Tumors

**Figure 5:**
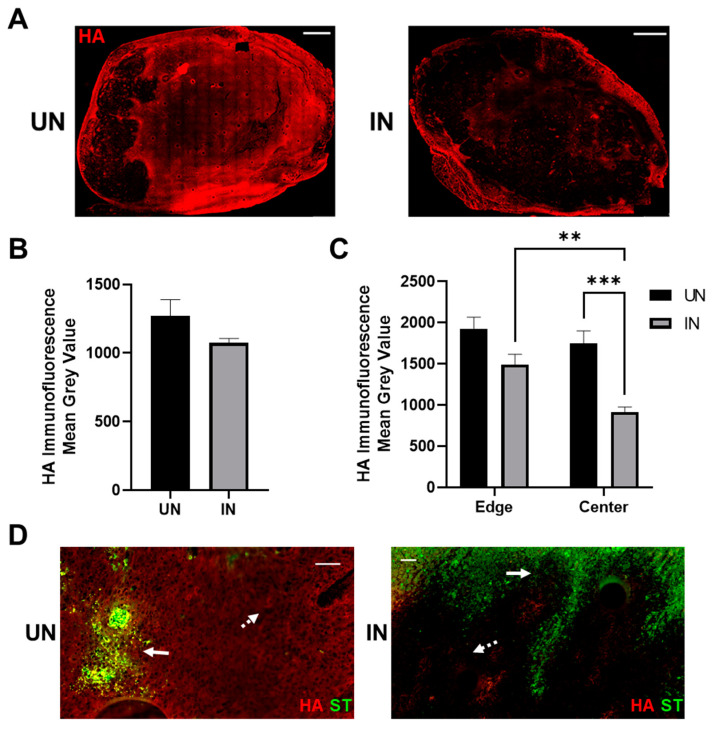
**Induction of YS-HAse leads to intratumoral HA depletion in vivo.** (**A**) IF staining of YS-HAse-treated tumors for HA. C57BL/6 mice bearing orthotopic EO771 tumors (~100 mm^3^) were i.v. administered 5 × 10^6^ cfu of YS-HAse per day over two days (*n* = 5). Forty-eight hours after the first dose, the groups were i.p. administered PBS (UN) or 150 mg L-arabinose (IN) and then euthanized 24 h post-induction. Serial tumor sections were then subjected to IF staining to detect HA. All images are representative of ≥3 experiments. Scale bar = 1000 µm. (**B**) Quantification of the total HA per tumor was performed using ZEN Blue software by dividing the HA channel MGV over the DAPI channel MGV to represent the total tumor area. (**C**) Quantification of the MGV of HA at the tumoral leading edges (~2 mm from the edge) and in the centers of UN and IN tumors was determined using ZEN Blue software. Values represent the average of 5 random areas each on the edges and centers. ** *p* < 0.01 and *** *p* < 0.001, two-way ANOVA with Sidak’s multiple comparisons test. (**D**) Overlay of the HA and ST channels on uninduced (UN) and induced (IN) tumors treated with YS-HAse. Solid arrows indicate ST; dashed arrows indicate distal HA. Scale bar = 50 µm.

To evaluate whether greater ST colonization is associated with intratumoral HA depletion, additional serial sections from Figure 4B were evaluated using HABP IF staining to visualize the intratumoral HA. UN YS-HAse-treated tumors maintained elevated and widespread levels of HA (Figure 5A, left), indicating that the HAse expression was tightly regulated. In contrast, the induction of YS-HAse resulted in a dramatic reduction of the HA signal, particularly within the tumor center (Figure 5A, right). The HA signal on the leading edges of the IN tumors remained elevated, with no significant difference between the UN and IN YS-HAse-treated tumors (Figure 5B). Comparisons of the HA IF signal from the tumor edges and centers (Figure 5C) yielded significant reductions in the HA signal when comparing the edges and centers of IN YS-HAse-treated tumors (*p* < 0.01) and centers of UN and IN YS-HAse-treated tumors (*p* < 0.001). Furthermore, the overlay of ST and HA signals revealed that HA degradation occurred distally from colonization in IN YS-HAse-treated tumors (Figure 5D), which is in line with the HAse secretion by YS-HAse. Overall, these results demonstrate that IN YS-HAse degrades intratumoral HA distally from colonization and is associated with increased tumor diffusion.

### 3.6. Systemically Delivered YS-HAse Controls Orthotopic BC Tumor Growth in Immunocompetent Mice

**Figure 6:**
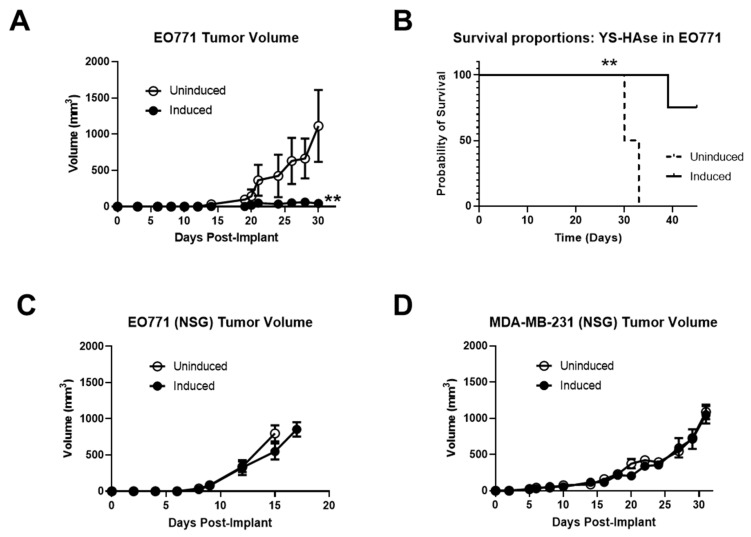
**YS-HAse controls EO771 tumor growth and improves survival in an immunocompetent murine model.** (**A**) C57BL/6 mice bearing orthotopic EO771 tumors (>50 mm^3^) were administered 5 × 10^6^ cfu per day of YS-HAse by i.v. injection on two consecutive days. Forty-eight hours after the first dose, the groups were i.p. administered PBS (uninduced) or 150 mg L-arabinose (induced) (*n* = 3). The tumors were measured every 2 to 3 days using digital calipers. ** *p* < 0.01, *t*-test. Mice were euthanized when the tumors reached 15 mm in diameter, and the overall survival (**B**) was recorded. ** *p* < 0.01, log–rank test. NSG mice bearing orthotopic EO771 (**C**) or MDA-MB-231 (**D**) tumors (>50 mm^3^) were treated with YS-HAse and UN/IN according to the same dosage and schedule as in (**A**) (*n* = 3).

Due to its ability to degrade intratumoral HA in vivo and the importance of HA in tumor progression, we hypothesized that the IN YS-HAse treatment could potentially retard tumor growth. Thus, female C57BL/6 mice bearing orthotopic EO771 tumors were i.v. administered YS-HAse daily for two days (1 × 10^7^ cfu total) and UN or IN via i.p. injection on day three. At two days post-i.p. injection, the tumor growth in UN YS-HAse-treated mice diverged from that of the IN tumors and began steadily increasing for the duration of the study (Figure 6A). In contrast, tumors of the IN YS-HAse treatment group showed significant control, steadily holding at a median volume of <60 mm^3^ (*p* < 0.01). By day 33 post-tumor implantation, all UN YS-HAse-treated mice had succumbed to disease, whereas all IN YS-HAse-treated mice remained alive (Figure 6B). These data strongly suggest that the induction of HAse expression and degradation of HA, in addition to enhancing ST colonization, also induces therapeutic effects in the EO771 breast cancer model.

In the desmoplastic tumor microenvironment (TME), HA-CD44 interactions influence innate immunity, playing a role in the activation and recruitment of various immune subsets [39,40]. Thus, we sought to determine whether the therapeutic effects of HA depletion seen in immunocompetent mice were recapitulated in immune-deficient mice. We first employed the orthotopic EO771 model in NOD/SCID/gamma (NSG) mice to directly assess the contributions of the immune system to tumor growth control elicited by IN YS-HAse treatment. Female NSG mice were orthotopically implanted with 1 *×* 10^5^ EO771 cells. When the tumors were palpable, the mice were administered YS-HAse, as previously indicated. The tumor growth was monitored over time until an endpoint of 15 mm in diameter was reached. As shown in Figure 6C, no significant difference in tumor growth was observed between UN and IN mice, with all tumors steadily increasing in size regardless of treatment.

To ascertain whether the lack of tumor growth control in treated NSG mice was limited to the EO771 mouse model, we repeated this study in an immune-deficient human xenograft model employing NSG mice and MDA-MB-231 cells. Female NSG mice were orthotopically implanted with 3 *×* 10^6^ MDA-MB-231 cells and treated with YS-HAse, as previously indicated. As shown in Figure 6D, the treatments with UN or IN YS-HAse did not control the tumor growth, despite its slower rate of growth compared to EO771 tumors in NSG mice. Indeed, both the UN and IN cohorts of mice experienced similar growth rates, such that the humane experimental endpoint was reached at 15–17 days for the EO771 group and 32 days for the MDA-MB-231 group. These results suggest that the tumor growth control observed upon treatment with IN YS-HAse is immune-dependent and is influenced by HA depletion.

### 3.7. YS-HAse Treatment Decreases Immune Subsets in Areas of HA Degradation

**Figure 7:**
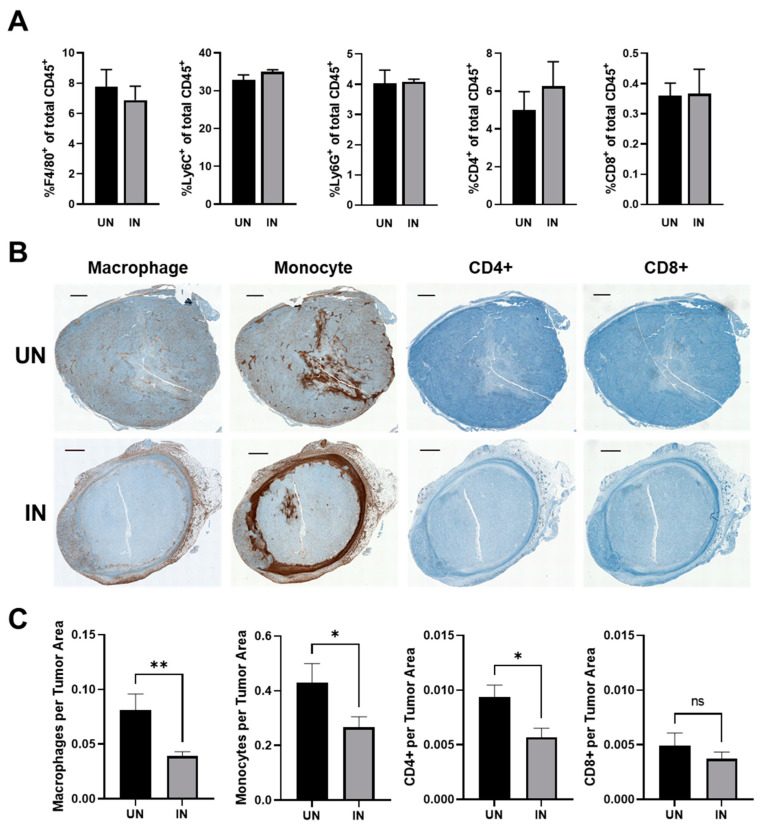
**YS-HAse treatment restructures the BC immune contexture by reducing the prevalence of potentially suppressive subsets within the tumor center.** (**A**) The flow cytometry analysis of YS-HAse-treated tumors. C57BL/6 mice bearing orthotopic EO771 tumors (~100 mm^3^) were administered 5 × 10^6^ cfu of YS-HAse by i.v. injection on two consecutive days. Forty-eight hours after the first dose, the groups were i.p. administered PBS (UN) or 150 mg L-arabinose (IN) and then euthanized 48 h post-induction. The tumor homogenates were then subjected to flow cytometry to evaluate the intratumoral immune phenotypes. (**B**) Immunohistochemical (IHC) staining of YS-HAse-treated tumors. C57BL/6 mice bearing orthotopic EO771 tumors (~100 mm^3^) were treated with YS-HAse and uninduced or induced as in (**A**). Twenty-four hours post-induction, the mice were euthanized, and serial tumor sections were subjected to IHC staining to detect murine macrophages, monocytes, and CD4+ and CD8+ T cells. All images are representative of ≥3 experiments. Scale bar = 1000 µm. (**C**) Quantification of immune subsets in the tumor centers was performed using QuPath software by defining the area of the tumor center and training a pixel classifier to distinguish between the tumor tissue and immune subsets. * *p* < 0.05 and ** *p* < 0.01, *t*-test.

Within desmoplastic tumors, HA has been shown to recruit and interact with macrophages and monocytes and to regulate their pro-tumorigenic activity [40,41]. The primary receptor for HA, CD44, has also been identified as a regulator of T cells [39]. We therefore investigated the effect of YS-HAse treatment on the intratumoral levels of macrophages, monocytes, and CD4+ and CD8+ T cells by flow cytometry and IHC. EO771 tumors from mice treated with YS-HAse were processed into single-cell suspensions and subjected to antibody staining for multicolor flow cytometry. An analysis of the tumor samples showed no significant differences in the percentages of the cells positive for the selected immune markers, including F4/80, Ly6C/G, CD4, and CD8, between tumors treated with UN and IN YS-HAse (Figure 7A). An analysis of the cells positive for NK1.1, as well as CD8^+^NK1.1^+^ cells, further showed no significant differences between the tumor treatments (Supplementary Figure S2A). Additionally, no significant difference was observed in the percentage of cells positive for PD-1 and the selected immune markers between the treatment groups (Supplementary Figure S2B). Ultimately, these data suggest that the IN YS-HAse treatment did not modify the frequency of these intratumoral immune subsets.

Whereas the frequency of the intratumoral immune subsets may not change, the IN YS-HAse treatment may restructure the immune contexture (spatial organization) within the tumors as a result of greater central HA depletion and vector colonization. To explore this, serial sections of YS-HAse-treated EO771 tumors were stained with primary antibodies against macrophages, monocytes, and CD4+ and CD8+ T cells, followed by DAB with hematoxylin counterstaining. The tumors from mice treated with UN YS-HAse contained elevated macrophages and monocytes across the entire tumor section, whereas the centers of the tumors treated with IN YS-HAse exhibited a marked decrease in these immune subsets (Figure 7B). Comparing the frequency of each subset found within the tumor centers, we observed a significant decrease in macrophages (*p* < 0.01) and monocytes (*p* < 0.05) (Figure 7C). Additionally, a significant decrease in CD4+ T cells (*p* < 0.05) was observed in the tumors treated with IN YS-HAse, whereas no significant change was observed with the CD8+ T cells. Overall, these data suggest that the IN YS-HAse treatment restructures the immune contexture within BC tumors through HA degradation, resulting in a more conducive microenvironment for the pre-existing antitumor immune milieu.

## 4. Discussion

Many solid cancers, including breast cancer, contain elevated levels of HA that are correlated with a poorer prognosis [6]. Due to its pro-tumorigenic activities, including the regulation of pro- and anti-inflammatory cytokine production, pro-survival signaling, and acting as a physical barrier to therapeutics, HA remains an attractive target for improving therapeutic efficacy in solid tumors [19]. Previous attempts to degrade intratumoral HA using mammalian HAses have been largely unsuccessful, as their systemic activity and broad substrate specificity have resulted in severe adverse effects, particularly within the musculoskeletal system [19,21,22,42]. Most notably, a phase II trial studying the addition of a pegylated form of recombinant human HAse (PEGPH20) with the chemotherapeutic treatment FOLFIRINOX was halted due to the detrimental effects of PEGPH20, including increased thromboembolic events, myalgias, and overall grade 3 to 4 toxicity [21]. The development of a well-tolerated and tumor-specific HAse is therefore a crucial and unmet need that could potentially be met with YS-HAse.

We previously developed an HAse-expressing ST (bHs-ST) using the strain χ8768, which showed success in degrading intratumoral HA within pancreatic tumors [43]. In contrast with the YS1646 strain, χ8768 tightly anchors the expressed HAse to its membrane and was selected for this trait to prevent potential off-tumor targeting. However, this limited HA degradation to the areas of ST colonization, possibly restricting its efficacy in vivo. The safety of χ8768 itself is an additional barrier, as dose-limiting toxicity was evident in our experimental mice, and this strain has not yet been evaluated in human subjects. Thus, we explored the use of YS1646 as a safer vector for HAse delivery due to its excellent tolerance in cancer patients [24,25]. Indeed, the mice tolerated YS-HAse dosages four-fold higher than bHs-ST, with no observable ill effects regardless of the immune status. Whereas the depletion of HA was not evaluated in healthy tissue, mice did not exhibit impaired mobility following the treatment, in contrast with frequent musculoskeletal adverse effects following systemic PEGPH20 administration [22]. Furthermore, upon the discovery of HAse secretion by YS-HAse, we theorized that the treatment with YS-HAse would enable intratumoral HA degradation distal from the sites of ST colonization, in contrast to bHs-ST. We observed that, in addition to the robust degradation of intratumoral HA in areas of colonization, HA degradation was evident further from the sites of colonization. Thus, these areas of distal degradation may potentiate greater ST diffusion, enabling a greater spread of ST-based or standard-of-care therapeutics across larger areas of viable tumor tissue.

Within tumors, HA is known to accumulate at the “leading edge”, a somewhat arbitrary term describing the outer periphery of the tumor tissue and so-called due to the tumor’s ability to invade and expand from these edges [44]. We have shown that, while YS-HAse was able to significantly degrade HA in the tumor centers upon induction, the HA within these leading edges remained. The remaining HA was present at these edges in such amounts that the quantification of the total HA was not significantly different between tumors treated with UN and IN YS-HAse but was significantly depleted upon induction when considering only the tumor centers. The lack of HA degradation on the leading edges may be due to the manner in which ST colonize tumors; they follow chemoattractants emanating from necrotic tumor centers and preferentially colonize and proliferate within these areas and therefore would begin HA degradation from the center [26]. As such, it is possible that an analysis of HA degradation at later timepoints may show a further depletion of leading-edge HA. Whereas the inability to degrade this HA may be viewed as a potential source of further tumoral spread, this was not observed in our in vivo studies; indeed, we observed significant tumor growth control and improved survival compared to the UN cohort, suggesting that the elimination of leading-edge HA may not be necessary for therapeutic benefit.

Previous studies and clinical trials targeting intratumoral HA have shown that HA degradation alone is unable to prevent tumor growth or metastasis and must be paired with an existing therapeutic to prompt any benefit [22,42]. However, many of these studies employed mammalian HAses that were constitutively active and thus required reduced doses to avoid adverse effects, which may have limited their potential efficacy. Additionally, some studies have suggested that it is the combination of HA and HAse activity, specifically HYAL1, rather than HA alone that promotes metastasis through the creation of fragmented HA that can induce CD44 cleavage [37,45]. These fragments have also been implicated in promoting tumor angiogenesis in bladder, prostate, and head and neck carcinomas [37,46]. Despite these observed tumor-potentiating qualities, we have not seen such effects upon treatment with YS-HAse.

Many pro-tumorigenic activities of HA can be attributed to its interactions with CD44, particularly with regards to immune subset recruitment, activation, and association [39,40]. Specifically, HA-CD44 interactions have been implicated in the recruitment and activation of T cells [4], monocytes [47], and macrophages [11,48] that promote an immunosuppressive TME. In BC, high levels of macrophages have been correlated with HA accumulation, and both phenotypes are associated with a worse prognosis [8,48]; the macrophage levels in BC stroma were additionally shown to decrease following disruption of HA-CD44 interactions [11]. Additionally, CD4+ and CD8+ T cells were noted to have opposing roles in BC progression and outcome, with the former associated with negative prognostic effects [49]. Thus, the significant decrease in macrophages, monocytes, and CD4+ T cells, but not CD8+ T cells, in tumor centers following central HA degradation highlights both the importance of HA in altering the intratumoral immune repertoire and the effects of YS-HAse treatment in disrupting these interactions. Cytokines and chemokines such as CXCL10 [39], IL-12 [40], IL-17 [50], and IFN-γ [47] are additionally implicated in activating these immune subsets; however, the flow cytometric data revealed no significant differences in the immune subset activation between treatment conditions. These data may further reflect how YS-HAse treatment restructures the spatial organization of the immune subsets within the TME but does not alter the subset frequency. Future work to characterize the immune subsets migrating outside the area of HA degradation and their cyto- and chemokine interactions may provide greater insight into the tumor growth control observed in the immunocompetent, but not immune-deficient, mice administered IN YS-HAse.

Whereas YS-HAse may be therapeutic on its own, we anticipate that it may provide further benefits when used in combination with micro- or macromolecule-sized therapies that were previously unable to passively diffuse into solid tumors. To maximize this diffusion, YS-HAse could also be paired with additional ECM-degrading agents, such as those targeting collagen. Furthermore, the YS1646-based system described here may be employed to develop novel tumor-targeted agents for a variety of other cancers that may be susceptible to enzymatic degradation of the stromal components critical for growth, survival, and invasion.

## 5. Conclusions

In this study, we developed YS-HAse, an ST system that is novel in its secretion of a bacterial HAse and employs a clinically tested and well-tolerated strain of ST. The expression of HAse is tightly controlled through an inducible promoter system, thus eliminating the off-tumor effects previously elicited by constitutive HAses. YS-HAse can effectively degrade HA in vitro and in vivo, and its use in an orthotopic murine model of BC resulted in a significantly improved tumor growth control and survival. Its mechanism of action is immune-dependent, as exhibited by its inability to provide a therapeutic benefit in immunocompetent murine models. Overall, YS-HAse may serve as an effective treatment alone or as a spreading agent for existing anticancer therapeutics.

## Data Availability

No new data were created or analyzed in this study. Data sharing was not applicable to this article.

## Supplementary Materials

The following supporting information can be downloaded at https://www.mdpi.com/article/10.3390/cancers14194614/s1: Figure S1: Tumor-specific colonization by YS1646 persists 7 days post-treatment. Figure S2: YS-HAse treatment does not significantly decrease the tumor-potentiating immune subsets in whole tumors.
