# Supplementary material for: Targeted Depletion of Hyaluronic Acid Mitigates Murine Breast Cancer Growth
Source: Cancers (Basel). 2022 Sep 23;14(19):4614. doi: 10.3390/cancers14194614 (PMC9562634; doi:10.3390/cancers14194614)
Supplement: Supplementary file 1 [file cancers-14-04614-s001.zip › Supplemental proofed.pdf]

Days Post-Injection:

7

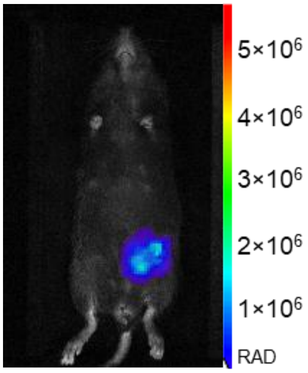

Figure S1. Tumor-specific colonization by YS1646 persists 7 days post-treatment. C57BL/6 mice bearing orthotopic EO771 tumors (>150 mm^3) were administered $5x10^6$ colony-forming units (cfu) of YS1646 expressing luciferase (YS-LUX) by intravenous (i.v.) injection. Intravital imaging was performed over several days using IVIS. Representative image shown.

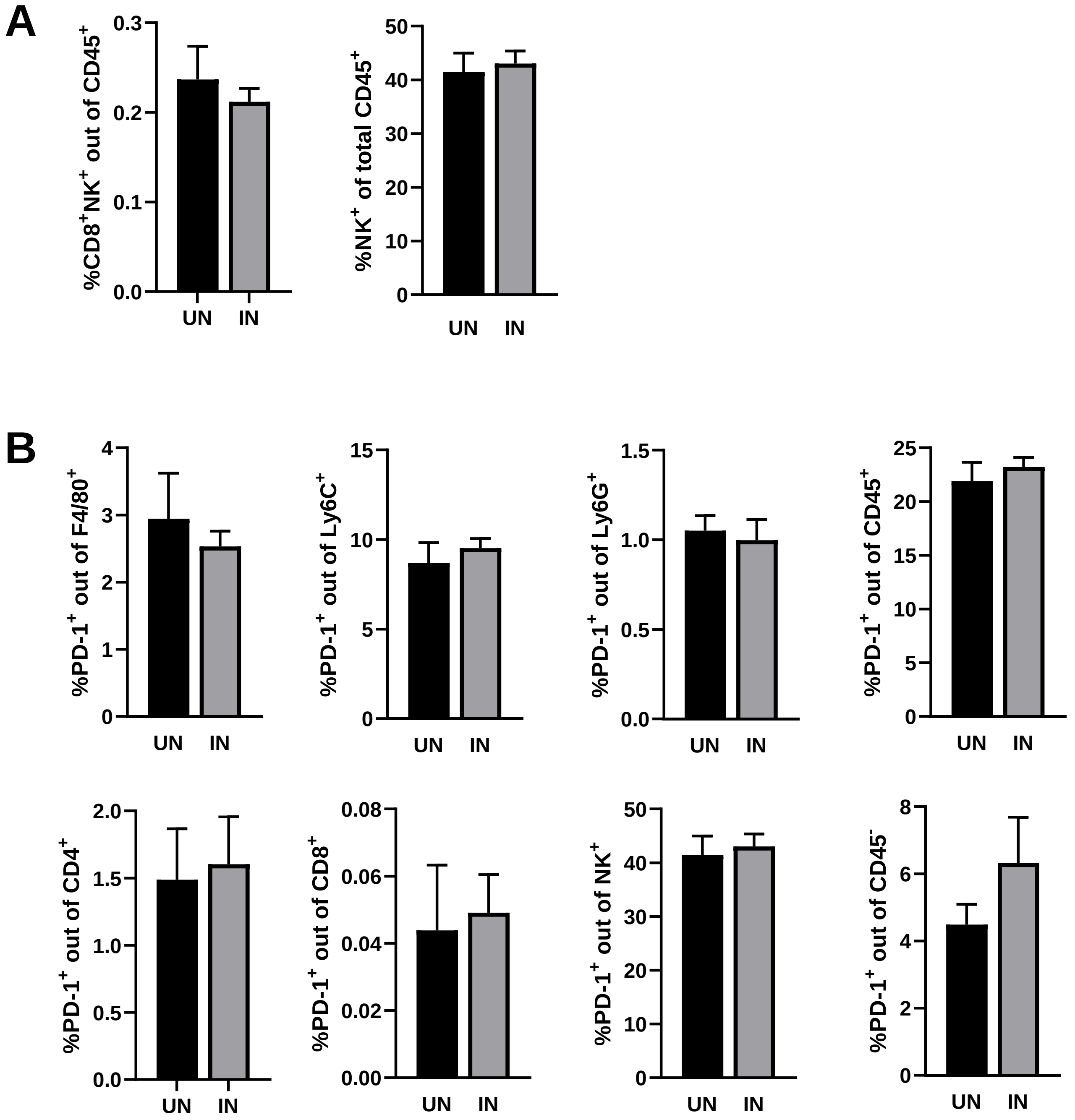

Figure S2. YS-HAse treatment does not significantly decrease tumor-potentiating immune subsets in whole tumors. **(A)** C57BL/6 mice bearing orthotopic EO771 tumors (~100 mm^3) were administered 5x10^6 cfu of YS-HAse by i.v. injection on two consecutive days (*n*=3). Forty-eight hours after the first dose, groups were i.p. administered PBS (UN) or 150 mg L-arabinose (IN) and then euthanized 48 hours post-induction. Tumor homogenates were subjected to flow cytometry to evaluate intratumoral immune phenotypes. Percentages of cells positive for immune markers out of all CD45-positive cells were quantified. **(B)** Out of cells positive for various immune markers, percentages of PD-1-positive cells were quantified.
